# Move Your Body toward Healthy Aging: Potential Neuroprotective Mechanisms of Irisin in Alzheimer’s Disease

**DOI:** 10.3390/ijms241512440

**Published:** 2023-08-04

**Authors:** Tatiani Bellettini-Santos, Hemily Batista-Silva, Clairton Marcolongo-Pereira, Fernanda Cristina de Abreu Quintela-Castro, Rafael Mazioli Barcelos, Kelly Cristina Mota Braga Chiepe, Joamyr Victor Rossoni, Roberta Passamani-Ambrosio, Bruno Spalenza da Silva, Orlando Chiarelli-Neto, Michelle Lima Garcez

**Affiliations:** Graduate Program of Research and Extension (CEPEG), University Center of Espirito Santo, Espírito Santo 29703-858, Brazil; tbsantos@unesc.br (T.B.-S.); hbsilva@unesc.br (H.B.-S.); cmpereira@unesc.br (C.M.-P.); fernanda.castro@unesc.br (F.C.d.A.Q.-C.); rafael.barcelos@unesc.br (R.M.B.); kchiepe@unesc.br (K.C.M.B.C.); jvrossoni@unesc.br (J.V.R.J.); rpambrosio@unesc.br (R.P.-A.); brunosilva821@hotmail.com (B.S.d.S.); ochiarelli@unesc.br (O.C.-N.)

**Keywords:** Alzheimer’s disease, apoptosis, autophagy, cell death, exercise, irisin, neuroinflammation, neuroprotection, oxidative stress

## Abstract

Alzheimer’s disease (AD) is the leading cause of dementia in older adults, having a significant global burden and increasing prevalence. Current treatments for AD only provide symptomatic relief and do not cure the disease. Physical activity has been extensively studied as a potential preventive measure against cognitive decline and AD. Recent research has identified a hormone called irisin, which is produced during exercise, that has shown promising effects on cognitive function. Irisin acts on the brain by promoting neuroprotection by enhancing the growth and survival of neurons. It also plays a role in metabolism, energy regulation, and glucose homeostasis. Furthermore, irisin has been found to modulate autophagy, which is a cellular process involved in the clearance of protein aggregates, which are a hallmark of AD. Additionally, irisin has been shown to protect against cell death, apoptosis, oxidative stress, and neuroinflammation, all of which are implicated in AD pathogenesis. However, further research is needed to fully understand the mechanisms and therapeutic potential of irisin in AD. Despite the current gaps in knowledge, irisin holds promise as a potential therapeutic target for slowing cognitive decline and improving quality of life in AD patients.

## 1. Introduction

Alzheimer’s disease (AD) is currently the most common cause of dementia among older adults, as it accounts for 60–80% of dementia cases [1]. The global estimated number of patients with dementia was 57.4 (50.4–65.1) million in 2019, and this figure is expected to increase to 152.8 (130.8–175.9) million by 2050 [2]. Therefore, the huge burden of AD in terms of prevalence and societal costs has led to a worldwide call for action. 

AD leads to the loss of cognitive functioning, impact thinking, remembering, reasoning, and behavioral abilities, which impair daily activities. Dementia eventually progresses to the most severe stage, causing complete dependency on others for help with the basic activities of daily living [3].

There is no cure for AD; the treatments only delay its progress or symptoms. The treatments available are galantamine, rivastigmine, and donepezil, which are cholinesterase inhibitors that increase the overall amount of acetylcholine in the brain and are prescribed for mild-to-moderate AD. Memantine, which is an N-methyl-D-aspartate (NMDA) antagonist, prevents glutamate excitotoxicity in moderate-to-severe stages [4]. Recently, the Food and Drug Administration (FDA) approved (accelerated approval) lecanemab and aducanumab, which are immunotherapies that target the protein beta-amyloid to reduce amyloid plaques, which are one of the main features of AD. However, although clinical studies using lecanemab and aducanumab have shown a reduction in the levels of amyloid in patients’ brains, and the ability of these medications to slow cognitive decline is still inconsistent [5,6].

In view of this scenario, further research is necessary to slow the cognitive impairment and improve the quality of life of these patients. Additional immunotherapy and other drug therapies, cognitive training, diet, and physical activity are some of the alternatives proposed by recent studies.

**Figure 1:**
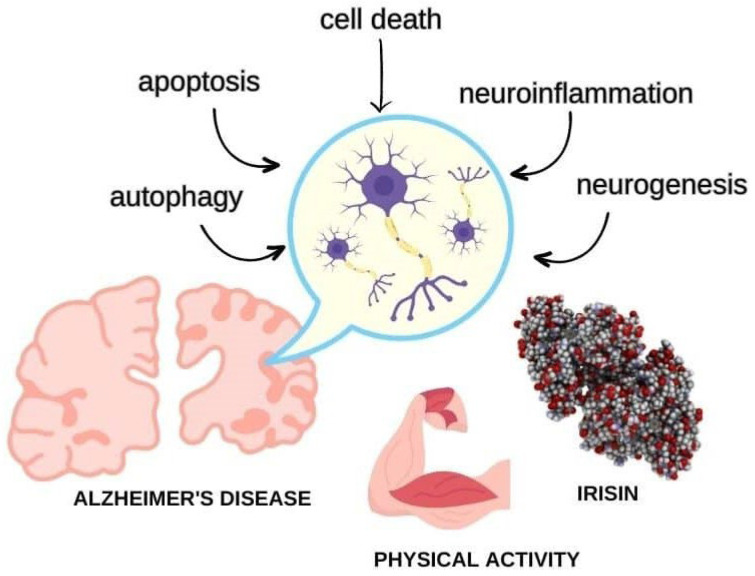
Irisin is a hormone produced in response to physical activity that has potential to treat Alzheimer’s disease by regulating synaptic function, modulating autophagy, inhibiting cell death pathways, and mitigating oxidative stress and neuroinflammation to promote neuroprotection and slow the progression of dementia.

Physical activity is protective of brain function, and most of the risk factors for AD are diseases related to a sedentary lifestyle (i.e., obesity, diabetes, dyslipidemia, and atherosclerosis). The relationship between improving cognition and preventing AD through physical activity has been extensively investigated [7]. Even though the studies regarding physical activity and cognitive decline have convergences concerning the protocol, duration, stage of cognitive decline, and other methodological biases, most of them have shown a positive effect in terms of preventing and slowing cognitive impairment in patients with dementia and AD. Consequently, the question to be answered is as follows: how does exercise act in the brain to prevent cognitive decline? Therefore, scientists discovered a hormone called irisin, which has the ability to drive the cognitive benefits of exercise and, hence, could hold great promise for preventing cognitive decline in AD patients. In the next section, we will discuss the potential benefits of irisin and the known mechanisms of irisin on the brain and AD (Figure 1).

## 2. Irisin

The fibronectin type III domain-containing protein 5 (FNDC5)/Irisin system is an essential regulator of energy metabolism. Irisin is a cleavage product of FNDC5 and acts as a myokine, as it has pleiotropic effects on peripheral tissues and the brain [8,9]. Understanding the normal physiology of this system is crucial to elucidating its role in maintaining metabolic homeostasis.

FNDC5 is a type I transmembrane protein composed of 209 amino acids. It contains an N-terminal signal peptide, a fibronectin type III domain, and a C-terminal hydrophobic transmembrane domain. The extracellular region of FNDC5 is proteolytically cleaved to release the active form of irisin [10].

The cleavage of FNDC5 occurs via a pro-protein convertase enzyme, furin, or a furin-like enzyme, resulting in the release of the N-terminal fragment, i.e., irisin. The precise mechanism and regulation of this cleavage process require further investigation [11]. After cleavage, irisin is released into the bloodstream. The mechanisms that underlie its release remain partially understood. It is hypothesized that irisin may be secreted via vesicular transport, such as exosomes or microvesicles, or via passive diffusion through the plasma membrane [12].

The FNDC5 gene is predominantly expressed in skeletal muscle tissue, but its expression can also be detected in other tissues. The expression of FNDC5 is regulated by several factors, including the peroxisome proliferator-activated receptor gamma coactivator-1 alpha (PGC-1α), exercise, and various signaling pathways, such as AMP-activated protein kinase (AMPK) and p38 mitogen-activated protein kinase (MAPK) [8,13].

Irisin has been detected in the brain, suggesting it has the ability to cross the blood–brain barrier (BBB). The exact mechanism of irisin transport across the BBB has not been fully elucidated. It is postulated that irisin may utilize transporters or receptors expressed through brain endothelial cells to enter the central nervous system [14]. Irisin exerts a wide range of effects on peripheral tissues and the brain. In peripheral tissues, irisin promotes browning of white adipose tissue, enhances glucose uptake, modulates lipid metabolism, and improves insulin sensitivity [8,9]. In the brain, irisin exhibits neuroprotective effects, enhances neurogenesis, and modulates synaptic plasticity [15,16].

The receptors mediating the physiological effects of irisin in peripheral tissues and the brain have not been definitively identified. However, recent studies suggest that irisin may interact with the αV integrin receptor or specific G protein-coupled receptors (GPCRs) to elicit its downstream signaling cascades [17]. Previous studies did not find any toxic effect of exogenous intervention using irisin on the health of murine models, and the optimum dose varies depending on the murine type and the induced model [18]. Regarding the pharmacokinetics of irisin, when injected into mice, it exhibits a short half-life of approximately 1 h [19]. Hence, the variability in its pharmacological activity and blood measurements may be attributed, at least in part, to its short half-life.

In addition to exercise and exogenous administration, various clinical and pre-clinical studies have demonstrated that certain drugs can induce the stimulation of irisin production, including simvastatin [20], fenofibrate [21], metformin [22], sitagliptin [23] retinoic acid [24], and follistatin [25]. Therefore, irisin’s pharmacological perspectives are intriguing, and most research is in the early stages; however, there is great interest due to its potential role in various physiological and pathological processes, including neurodegeneration.

## 3. Irisin and AD

Scientists have made significant efforts to develop strategies to counteract the mechanisms that lead to neuronal damage, synapse failure, and memory impairment in AD. This impairment is probably related to failures and loss of synapses [26,27]; therefore, therapies that restore or preserve synaptic function and cognition are highly desired.

Recent studies have shown that irisin, which is a myokine stimulated via physical exercise derived from the precursor protein fibronectin type III domain-containing protein 5 (FNDC5), is reduced in the brains and cerebrospinal fluid (CSF) of humans with AD [28]. In addition, increasing brain or peripheral levels of FNDC5/irisin enables us to attenuate synaptic and memory impairments in mouse models of AD [15]. Additionally, irisin exerts various beneficial effects on the brain, including the induction of genes related to neuron growth and survival in the hippocampus, which is a region critical for memory [29]. Moreover, irisin is known to play crucial roles in the CNS [30], including improving memory and pro-inflammatory cytokines in AD models [15,31,32]. Previous studies have reported that irisin is observed in the cerebrospinal fluid (CSF) and hypothalamus [33], and FNDC5 is known to be highly expressed in glia (e.g., astrocytes and microglia) and neurons in various brain regions [14,34,35,36].

Irisin is a hormone produced by muscle tissue in response to physical exercise. Regular physical exercise, specifically aerobic exercise, may increase irisin and have preventive effects on AD [15]. Irisin plays an important role in regulating metabolism and communication between muscle tissue and other organs, including the brain. It can also stimulate the production of neuroprotective substances, such as brain-derived neurotrophic factor (BDNF), which plays a crucial role in neuron survival and function [8].

In addition, irisin is capable of inducing the expression of genes related to the growth and survival of neurons in the hippocampus, which is a region of the brain fundamental to memory and eventually affected by AD [8,13]. Its reinforcement, whether it occurs pharmacologically or through exercises, may, therefore, constitute a new therapeutic strategy that could protect or repair synaptic function and prevent cognitive decline in AD [15], improving cognitive function and brain plasticity and reducing the risk of developing dementia [16].

Irisin is primarily released during moderate-to high-intensity aerobic exercises. Exercises such as running, brisk walking, cycling, swimming, and dancing are known to stimulate the release of irisin to a greater extent [37]. When released during aerobic exercise, irisin triggers a process of converting white fat cells into brown fat cells, which are more metabolically active. This finding suggests that irisin may play a role in body weight regulation and energy metabolism [8,38].

These activities involve rhythmic and continuous movements that increase the demand for oxygen and promote higher energy expenditure. However, it is important to note that any form of physical exercise can have health benefits and promote the release of irisin, even if it does so to a lesser extent than more intense aerobic exercises [39].

The production of irisin is limited to specific tissues. The majority of this hormone is released into the peripheral bloodstream by skeletal muscles during exercise [8,37]. Other sources of irisin are subcutaneous and visceral adipose tissues [37]. Thus, irisin is not only secreted by skeletal muscles as a substance called myokine, but can also be released into the peripheral bloodstream as an adipokine. The release of irisin by adipose tissue occurs in a fashion complementary to its release by skeletal muscles during physical exercise, regulating cerebral energy metabolism and improving cerebral blood flow [15,37]. The potential specific mechanisms of irisin that contribute to neuroprotection will be further discussed in this paper.

Irisin was initially described as a myokine released by skeletal muscles following physical exercise associated with exposure to low temperatures, enabling an adaptive response of “browning” (conversion of white fat cells into brown fat cells) and thermogenesis [29]. In this regard [40]. Lee et al. (2014) conducted experiments to understand the interactions between physical exercise, cold exposure, and irisin. They compared irisin levels in 10 healthy adults after gradual exposure to cold and two standard stress tests: maximum exercise using a bicycle ergometer (VO_2_ max) and a submaximal test (VO_2_ 40%). Serum irisin levels showed an increase after maximal exercise after the first hour. After 60 min of submaximal exercise, irisin levels significantly increased. These results confirm the stimulating activity of exercise on irisin secretion. It was also observed that the temperature decrease can affect irisin secretion. The cooling protocol demonstrated that irisin levels were positively and directly related to the cold sensation experienced by the individuals under examination. The comparison between the data obtained in the cooling protocol and the exercise protocol showed that the increase in irisin was similar during both tests. However, the increase in energy expenditure was significantly higher during maximum exercise than cold exposure [40].

The dissociation between the changes in irisin levels and energy expenditure suggests that the signaling pathways are either not or only partially related to exercise and cold exposure. Confirming this relationship, a systematic review conducted by Flouris et al. (2017) [40,41] suggested that regular exercise alone cannot be considered a key factor in increasing irisin and uncoupling protein 1 (UCP1), which is a mitochondrial protein mainly found in brown fat cells (brown adipose tissue), as well as in smaller quantities in white fat cells (white adipose tissue); however, this effect may result from the synergistic action of low temperatures and physical exercise. Also in 2017, Coker et al. [42] measured the irisin serum concentrations of eight healthy adults during the Yukon Arctic Ultra event (430 miles during temperatures that ranged from −45 °C to −8 °C). The results indicated that cold exposure and extreme physical activity promoted a significant increase in serum irisin levels. This finding further reinforces the possible synergy between exercise and exposure to low temperatures [29].

However, it is important to highlight that there are still many aspects to be clarified and investigated, such as the precise detection of irisin levels in plasma, pharmacokinetic and pharmacodynamic profiles, and modulation related to different pathological conditions [15,37]. A deeper understanding of the irisin pathway and the identification of new pharmacological tools and molecules that act on this target are matters of great importance and relevance to biomedical research. Moreover, the conflicting results observed in recent studies suggest the need for further investigation to elucidate the relationship between physical exercise, cold exposure, and irisin in different contexts and conditions. In agreement with Maak et al. (2021), we conclude there are hundreds of studies reporting increased irisin levels in response to different physiological challenges that are compromised by the use of flawed quantitative assays [43].

## 4. Autophagy

Autophagy is a process of cellular self-degradation and recycling that protects cells from varying insults, including misfolded and aggregated proteins and damaged organelles, which is crucial to neuronal survival [44].

The modulation of autophagy, however, is a complex process, and the timing and extent of autophagy induction must be carefully controlled. For example, autophagic disorders may cause the blockage of autophagic flux, affecting cellular health [45] and increasing oxidative stress, inflammation, and AD progression [46,47].

**Figure 2:**
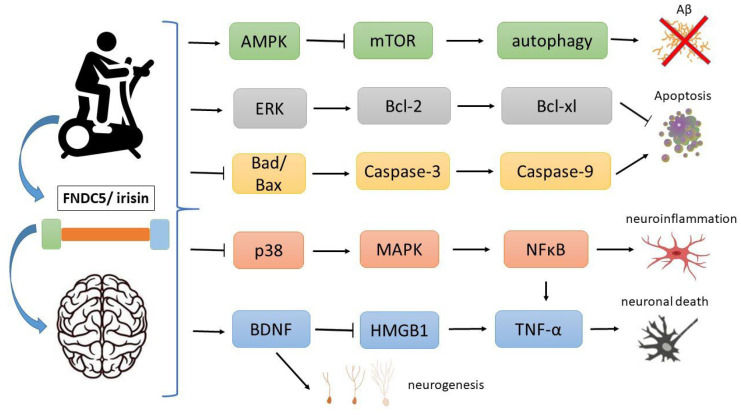
Potential mechanisms of irisin in Alzheimer’s disease (AD). Recent studies have shown that irisin, which is a hormone stimulated via physical exercise derived from the precursor protein fibronectin type III domain-containing protein 5 (FNDC5), is reduced in the brains and cerebrospinal fluid (CSF) of humans with AD. The AMP-activated protein kinase (AMPK) is a key pathway in autophagy. Irisin can modulate this pathway by activating the AMPK pathway and inhibiting mTOR, thus leading to the increase in autophagy and the clearance of amyloid and toxic aggregates in the brain. Irisin can decrease pro-apoptotic Bad, Bax and caspase-3, and caspase 9 while increasing anti-apoptotic proteins Bcl-2 and Bcl-xl through the ERK signaling pathway. Irisin demonstrates the inhibition of the necroptotic signaling pathway through increasing BDNF (which increases neurogenesis), mitigating the impact of HMGB1 and suppressing tumor necrosis factor (TNF-α) mRNA expression. It can also inhibit p38, STAT3, and NFκB, reducing neuroinflammation.

On the other hand, autophagic homeostasis could have beneficial effects in AD [48]. The activation of autophagy pathways has been shown to promote the clearance of protein aggregates and reduce their toxic effects [49]. The AMP-activated protein kinase (AMPK) is a key pathway in autophagy. Irisin can modulate this pathway by activating the AMPK pathway and inhibiting mTOR, thus regulating the AMPK/mTOR signaling pathway involved in autophagy [50,51]. The inhibition of mTOR might lead to an increase in autophagy and the clearance of amyloid and toxic aggregates in the brain (Figure 2).

Thus, there is ongoing exploration of pharmacological interventions and modulation of autophagy-related signaling pathways, including irisin, with the aim of stimulating autophagy as a potential therapeutic strategy [52]. However, the precise roles of irisin and autophagy in AD are yet to be investigated and remain unclear.

## 5. Cell Death

One of the hallmarks of AD is the accumulation of amyloid beta (Aβ) plaques and tau tangles in the brain, which trigger neuroinflammation and neuronal death [53,54,55].

Cell death has diverse forms within the body and is linked to numerous diseases and conditions, including AD. Apoptosis, which is a form of programmed cell death, is the dominant mechanism of cell demise [56]. Nevertheless, several other forms of cell death coexist, including necrosis, ferroptosis, pyroptosis, and necroptosis [57].

Necrosis is a particular type of cell death that stems from significant injury or stress, which can notably contribute to the depletion of brain tissue and impair function in AD. This form of cell death arises when cells are subjected to intense external damage, including the swelling of organelles, which is referred to as oncosis; the rupture of the plasma membrane; and the subsequent discharge of intracellular contents that possess pro-inflammatory properties [58].

Irisin has been shown to have beneficial effects on neuroprotection, exerting a protective effect against necrosis. This effect is accomplished by promoting the expression of brain-derived neurotrophic factor (BDNF), which is a growth factor that aids in the survival and adaptability of neurons [37,59]. A decrease in the serum levels of BDNF has been observed in patients with Alzheimer’s disease. However, exercise has been shown to provide dual benefits by increasing the expression of irisin and effectively improving cognitive dysfunction. These positive effects on cognition have been linked to the up-regulation of BDNF, establishing a significant association [60]. Moreover, irisin demonstrates a remarkable ability to inhibit the necroptotic signaling pathway by suppressing tumor necrosis factor (TNF-α) mRNA expression and mitigating the impacts of MCP1 and HMGB1 on downstream targets. Additionally, it exerts a potent anti-inflammatory effect by reducing the levels of pro-inflammatory cytokines and possesses significant antioxidant properties, which lead to neuroprotection [61].

In addition, irisin has the potential to improve insulin resistance and promote glucose homeostasis through the activation of PI3K/Akt signaling and p38 mitogen-activated protein kinase (p38MAPK) pathways [59]. Experiments conducted on animals demonstrated that the increased expression of FNDC5/irisin played a significant role in improving insulin resistance and reducing blood glucose levels in mice that were fed a high-fat diet [59]. In another experiment, mouse hepatocytes cultured with irisin under high glucose conditions were treated, and the results confirmed that irisin had the ability to reduce intracellular insulin resistance, enhance glycogen synthesis, and promote cell survival [62]. These pathways are impaired in AD and contribute to low glucose metabolism and neuronal death (Figure 2). As a result, irisin may play a crucial role in protecting patients against neuronal death and AD progression.

## 6. Apoptosis

Irisin has multiple effects on apoptotic pathways in different contexts. For example, in type 2 diabetes, it can decrease the pro-apoptotic proteins Bad, Bax, and caspase-3 while increasing the anti-apoptotic proteins Bcl-2 and Bcl-xl [63]. Similarly, in AD, there is insulin resistance and lowered glucose metabolism in the brain, which can have implications for apoptosis.

In addition, there are recognized vascular abnormalities and brain endothelial dysfunction in AD. Irisin was shown to improve endothelial dysfunction in mice by increasing phosphorylation of endothelial nitric oxide synthase (eNOS) and its pathway-related proteins, like Akt and AMPKα [64]. Also, irisin protected against vascular endothelial dysfunction by modulating the ERK signaling pathway; down-regulating Bax, caspase 3, and caspase 9 expression; and up-regulating Bcl-2 anti-apoptotic protein expression in human umbilical vein endothelial cells [65].

Moreover, this hormone was able to decrease inflammation and neuronal apoptosis after intracerebral hemorrhage by increasing the expression of integrin αVβ5 (irisin receptor), p-AMPK (promotes microglial/macrophage polarization to the M2-phenotype), and Bcl-2, while IL-1β, TNF-α, myeloperoxidase (MPO), and BAX expression were lowered after intracerebral hemorrhage in the mouse model [66].

Thus, these irisin mechanisms can contribute to apoptosis regulation and protect against apoptosis in AD (Figure 2). However, studies of AD models and patients are necessary to confirm the anti-apoptotic mechanisms of irisin in AD.

## 7. Oxidative Stress

Oxidative stress plays a crucial role in the development of human diseases. Reactive oxygen species (ROS) and reactive nitrogen species (RNS), including the superoxide anion radical (O_2_•^–^), hydrogen peroxide (H_2_O_2_), hydroxyl radical (•OH), nitric oxide (NO), and peroxynitrite (ONOO^–^), frequently exceed the body’s antioxidant defense and repair capacity, resulting in increased damage to biomolecules, such as nucleic acids, lipids, and proteins. This issue can induce necrosis or apoptosis, thereby playing an important role in cell growth, differentiation, and death [67,68,69,70].

Oxidative stress can be defined as the consequence of an imbalance between the formation and elimination of free radicals by cells, which can lead to damage at various cellular levels. Its influence can be observed in cardiovascular, renal, respiratory, neurological, and cancer- and diabetes-related diseases [67,71,72]. In this context, oxidative stress has been recognized as a factor that plays a significant role in the aging process, as well as in the progression of various neurodegenerative diseases, including AD [68,72,73].

The presence of oxidative stress in AD is evidenced by the occurrence of elevated levels of oxidized proteins, advanced glycation end products (AGEs) [74], and products derived from oxidative stress, such as 4-hydroxy-2,3-nonenal (HNE), 3-nitrotyrosine, 8-hydroxy-2’-deoxyguanosine (8-OHdG), 8-hydroxyguanosine (8-OHG), acrolein, malondialdehyde, and F2-isoprostanes, which are present in patients with AD and transgenic animal models. HNE is capable of modifying proteins and generating various effects, including the inhibition of neuronal glucose and glutamate transporters [75,76,77,78], and it has been identified as one of the signals that up- or down-regulate the levels of microRNAs believed to be involved in the pathogenesis of AD [79].

Moreover, research has shown that the chronic neuroinflammatory process induced by the presence of amyloid plaques in AD results in the hyperactivation of microglial cells, which impairs mitochondrial functions, exacerbates the neurodegenerative process, and contributes to the augmented production and synthesis of ROS [80].

Aerobic exercise has been shown to improve cognitive function and plasticity, particularly in neurodegenerative disorders such as AD, as it mediated by irisin released during the contraction of muscle fibers [81,82]. Experiments conducted on mice subjected to a swimming protocol revealed better protection against memory deficits induced by Aβ aggregates and a reduction in FNDC5/irisin levels in the brain. Additionally, this protocol prevented Aβ-induced reductions in FNDC5/irisin mRNA and protein in the mouse hippocampus [15]. Irisin has been shown to cross the blood–brain barrier and stimulate hippocampal neurogenesis by increasing the expression of BDNF [83]. In addition, exercise and irisin reduce neuroinflammation and ROS levels in hippocampal neurons due to a reduction in inflammatory cytokine release [81]. The levels of FNDC5/irisin are controlled by PGC-1α, which is known to mediate synaptic function and neuroprotection and induce ROS. Therefore, PGC-1 and peroxisome proliferator-activated receptor gamma (PPAR-γ) were investigated in mice, and it was observed that the mRNA expression of both was reduced in the hippocampi of the animals [15].

The antioxidant potential of irisin has recently received more attention from researchers. It appears to prevent oxidative stress by modulating the signaling pathways of AMPK, PI3K, Akt, and eNOS [84]. Therefore, the therapeutic potential of exogenous irisin therapy involves protection against inflammation, oxidative stress, and apoptosis. However, there is a significant gap between the evidence and the actual effect of irisin in the context of AD.

## 8. Neuroinflammation

Neuroinflammation is one of the hallmarks of AD and plays a key role in the pathogenesis of this disease [85]. Neuroinflammatory mechanisms comprise the activation of microglia, i.e., the main immune cell type of the central nervous system (CNS) [86]. Microglia play a complex role in the course of AD due to their diverse phenotypes, which have different morphological and functional characteristics [85]. In their steady state, microglia exhibit a branched shape with multiple thin processes through which they constantly monitor the CNS parenchyma. In response to pathological and inflammatory conditions, microglia change to an amoeboid morphology with increased phagocytic activity and the production of the pro-inflammatory cytokines interleukin IL-1β, IL-6, and TNF-α [87,88]. Moreover, microglia also release chemokines that are chemoattractants for peripheral immune cells to migrate to the CNS and produce ROS, NO, and prostaglandins [89].

In addition to microglia, astrocytes also strictly cooperate in the neuroinflammatory process. Indeed, inflammatory factors released by microglia may induce pro-inflammatory astrocytes [90]. Interestingly, both microglia and astrocytes may display pro-inflammatory (or neurotoxic) and neuroprotective phenotypes, which contribute to Aβ accumulation in AD [91]. Microglial activation and astrocyte metabolism dysfunction may result in the failure of Aβ clearance in the brain, which is an important factor in the progression of AD [92]. The microglia that surround Aβ plaques are usually of the neuroprotective phenotype in early AD, but change to the neurotoxic (pro-inflammatory) phenotype in the advanced stages [93]. Furthermore, “inflammaging”, which is characterized as a widely accepted hypothesis that aging is accompanied by a regulation of certain pro-inflammatory responses, is considered to be a “prodrome” for AD and microglial malfunction [94].

The modulation of neuroinflammation may be an important therapeutic target in AD. Physical activity was found to be a non-pharmacological anti-inflammatory agent that acts on multiple non-skeletal targets. The myokine FNDC5/irisin produced in response to physical exercise has been shown to protect various tissues through anti-inflammatory, anti-oxidative, and anti-apoptotic effects [84]. Interestingly, FNDC5/irisin exerts neuroprotective and anti-inflammatory effects in the brain, which mainly occur in the hippocampus, improving cognitive functions and neuronal plasticity [89], which are both functions affected in AD [95]. FNDC5/irisin levels have been reported to be reduced in the hippocampus and CSF in experimental models of AD. However, peripheral FNDC5/irisin overexpression rescued memory impairment in APP/PS1 ΔE9 mice, whereas peripheral or cerebral FNDC5/irisin blockade attenuated the neuroprotective actions of physical exercise on synaptic plasticity and memory, making FNDC5/irisin a novel agent capable of opposing synaptic failure and memory impairment in AD [15]. The therapeutic potential of FNDC5/irisin against dementia may involve inflammation, oxidative stress, and apoptosis [84,96].

It has been shown that co-treatment with irisin reversed cognitive damage and inhibited neuroinflammation in the hippocampi of diabetic mice induced by streptozotocin by reducing IL-1β and IL-6 levels, in addition to inhibiting the activation of p38, MAPK, and NFκB proteins [96]. In addition, irisin (100 µg/kg weekly i.p.) at pre-symptomatic age was able to reduce TNFα levels in the hippocampi and serum of female htau mice [32]. Furthermore, intracerebral hemorrhage is another condition in which irisin has demonstrated its anti-inflammatory effect. The treatment with irisin inhibited microglia and macrophage pro-inflammatory polarization and promoted anti-inflammatory polarization, in addition to inhibiting neutrophil infiltration in intracerebral hemorrhage. Mechanistically, the treatment with irisin significantly increased the expression of integrin αVβ5, p-AMPK and Bcl-2, as mentioned before, as well as decreasing the expression of IL-1β, TNF-α, and myeloperoxidase [66].

Furthermore, the mechanism via which FNDC5/irisin promote neuronal cell survival and synaptic plasticity may be associated with up-regulated BDNF levels [97]. It has been shown that BDNF expression in primary cortical neurons of mice after physical exercise is stimulated through the proliferator-activated receptor gamma coactivator 1-alpha (PGC1α)/FNDC5/irisin pathway [13]. Also, a cohort study demonstrated that the irisin levels in the CSF were positively correlated with BDNF, Aβ42, and cognitive status in AD patients [98]. Interestingly, the authors discovered that decreased CSF irisin was correlated with BDNF and Aβ42 levels, but not with tau protein. 

Moreover, advances in research have shown many physical exercise-related advantages for neurogenesis that involve increased BDNF levels in AD mouse models [99]. Nowadays, it is well known that adult hippocampal neurogenesis (AHN) is seriously affected by AD onset. Moreover, 5 × FAD transgenic mice, which constitute a mouse model of AD, were shown to induce AHN, but they only improved AHN without reducing Aβ plaques or improving memory. On the other hand, along with physical exercise, memory improvement, an increase in AHN, a reduction in Aβ plaques, and an increase in BDNF, IL-6, and *FNDC5* levels were observed [31].

Taking into account the fact that Aβ deposition contributes to the progression of neuroinflammation mediated via microglial activation, which leads to the loss of synapses and neurodegeneration [100], attenuation of Aβ-mediated deposition and accumulation may be the potential anti-inflammatory effect of irisin [89]. The role and importance of the anti-inflammatory effects of irisin in AD and the crosstalk between peripheral levels of irisin and its role in the CNS are becoming clearer, but further studies are needed to confirm their validity.

## 9. Conclusions

AD is a prevalent and devastating neurodegenerative disorder with no cure. Physical exercise itself has been associated with cognitive benefits and a reduced risk of developing AD. The irisin produced in response to physical activity has been shown to regulate synaptic function, modulate autophagy, inhibit cell death pathways, and mitigate oxidative stress and neuroinflammation, leading to neuroprotection. These mechanisms make irisin an intriguing candidate for interventions that aim to prevent cognitive decline and AD progression.

## 10. Future Directions

Irisin has potential for use in the prevention and treatment of AD; however, further research into animal models of AD is needed to understand the underlying mechanisms and establish effective therapeutic strategies. In addition, clinical studies with controlled protocols of physical activity of long duration, stratification of the stage of cognitive decline, and irisin measurement will be crucial in validating the therapeutic potential of irisin for AD pathology.

## Data Availability

Not applicable.
